# Probiotic Spores of *Shouchella clausii* SF174 and Displayed Bromelain Show Beneficial Additive Potential

**DOI:** 10.3390/ijms26030942

**Published:** 2025-01-23

**Authors:** Rowena Corona, Valeria Bontà, Loredana Baccigalupi, Ezio Ricca

**Affiliations:** 1 Gruppo Savio, 00071 Pomezia, Italy; rowena.corona@savioindustrial.it (R.C.); valeria.bonta@savioindustrial.it (V.B.); 2 Department of Molecular Medicine and Medical Biotechnology, Federico II University of Naples, 80126 Naples, Italy; lorbacci@unina.it; 3 Department of Biology, Federico II University of Naples, 80126 Naples, Italy

**Keywords:** *Bacillus clausii*, beneficial bacteria, antioxidants, surface display, functional probiotics

## Abstract

Probiotics have health-beneficial properties mainly due to either a direct action on the host or the modulation of the host microbiota. Health-beneficial properties have also been associated with a variety of plant-derived molecules, widely used as dietary supplements. This study explores the possibility of combining the actions of probiotics and of plant-derived molecules by developing beneficial, probiotic-carrying, heterologous molecules. To this extent, spores of SF174, a well-characterized probiotic strain of *Shouchella clausii* (formerly *Bacillus clausii*), were used to bind bromelain, a plant-derived mixture of endopeptidases with beneficial effects. Probiotic spores displaying bromelain maintained their antioxidant activity and acquired the endopeptidase activity of the heterologous molecule. The endopeptidase activity was stabilized by the interaction with the spore and largely preserved from degradation at simulated gastric conditions. Under conditions mimicking those encountered in the intestine, as well as upon spore germination, active bromelain was released from the spore surface. The *in vitro* results reported in this study support the idea that probiotics carrying beneficial heterologous molecules combine the health properties of the probiotic with those of the delivered molecule and pave the way for the development of a novel class of functional probiotics.

## 1. Introduction

The oral administration of live bacteria (probiotics) contributes to human health by acting directly on the host and/or indirectly, through the modulation of the gut microbiota composition [1,2,3,4,5]. Several species of bifidobacteria, lactobacilli, and bacilli have long been used as commercial probiotics [6,7,8] with strain-specific beneficial effects that include the reduction of gastrointestinal discomfort [1] and the improvement of immune health [9]. Probiotic preparations containing members of the *Bacillus* genus differ from all other probiotic products for the peculiarity of being administered as spores [10,11,12]. These are quiescent cell forms produced in response to nutrient limitations and adverse environmental conditions. Spores are extremely stable and can survive indefinitely in the absence of water and nutrients and in the presence of toxic chemicals, lytic enzymes, and conditions of extreme temperatures and pH [13]. In the presence of favorable conditions, spores germinate, originating vegetative cells able to grow and re-sporulate [14]. Therefore, the use of spores as probiotics takes advantage of their stability and resistance to ensure both an extremely long shelf-life for the product and safe transit through the stomach [10]. Once in the intestine, spores germinate and grow, temporarily colonizing that habitat and playing their beneficial roles [15].

More recently, it has been proposed that probiotics may be used to carry and deliver beneficial molecules to the host mucosal surfaces [16,17,18]. Such functional probiotics would then act synergistically with the carried molecules and combine the health properties of the probiotic with those of the delivered molecule [16,17,18]. In this context, the use of probiotic spores is particularly interesting, since spores efficiently adsorb heterologous molecules [19], stabilizing them under simulated gastric conditions [20].

This study explores the *in vitro* potentials of functional probiotic spores displaying bromelain, a complex mixture of cysteine endopeptidases purified from the pineapple plant (*Ananas comosus*) [21]. Bromelain has a long history in traditional medicine, and currently bromelain-containing products are widely commercialized as dietary supplements for their claimed beneficial effects against several diseases [21]. Indeed, bromelain has been reported to reduce pain and swelling after surgery or injury, ameliorate symptoms in osteoarthritis, digestive problems, and muscle soreness [21]. In addition, bromelain has been reported to have anti-inflammatory and antioxidant [22,23], anticancer [24], antithrombotic, and antifibrinolytic [25] activities. Although bromelain is stable in a wide pH range, it is seriously affected by the gastric conditions characterized by high acidity and the presence of gastric juices, which therefore limit the healthy effect of bromelain when ingested as a food supplement. To improve bromelain gastric stability and its biotechnological applications, several bromelain-immobilization strategies have been attempted [26]. Bromelain has also been immobilized on spores of a probiotic strain of the *Bacillus* genus, not characterized at the species level [27]. Spore-adsorbed bromelain was more stable than free bromelain at high temperatures (>60 °C) and low pH values (pH 3–4) [27]. In a different study of the same group, bromelain was immobilized on spores of a probiotic *B. cereus* strain (KY746353.1), isolated from an fermented African food [28]. In this case, spore-adsorbed bromelain increased the anti-inflammatory effects of free bromelain in a rat model of edema [28].

Here, bromelain was displayed on spores of the well-characterized probiotic strain SF174 of *Shouchella clausii* (formerly *Bacillus clausii*), a strain isolated from an ileal biopsy of a human healthy volunteer, found able to grow in anaerobic conditions [29] and shown to have a strong antioxidant activity *in vitro* [30]. In addition, cells of SF174 produce antimicrobial molecules active against cells of *Listeria monocytogenes* and *Candida albicans* as well as molecule(s) active against the biofilm produced by *Mycobacterium smegmatis* [31]. SF174 spores efficiently bound *in vitro* intestinal epithelial model cells [30] and *in vivo* had anti-inflammatory activity in a mouse [32] and a rat [33] model. In the mouse model of inflammation, SF174 spores modified the composition of the gut microbiota by increasing the relative abundance of beneficial intestinal bacteria, such as members of the *Bacteroidetes* and *Akkermansia* genera [32]. Purified spores of SF174 were shown to efficiently adsorb bromelain, and the spore-adsorbed molecules were more stable than free bromelain at simulated gastric conditions. At simulated intestinal conditions, as well as upon spore germination, enzymatically active bromelain was released from the spore surface, indicating the spore of SF174 as a valuable carrier for the delivery of bromelain to intestinal mucosal surfaces. The strong antioxidant activity of SF174 spores [30] was maintained and slightly increased when bromelain was displayed. 

This study was aimed at assessing whether probiotic spores could be manipulated to carry heterologous molecules acquiring specific properties of the carried molecule and retaining their own properties. Presented results indicate that SF174 spores efficiently bind bromelain, acquiring its endopeptidase activity and protecting it against degradation at simulated gastric conditions. Moreover, adsorbed spores maintain their antioxidant activity and the capacity to germinate, essential for their probiotic role. Reported results pave the way to the development of a new class of spore-based functional probiotics. 

## 2. Results

### 2.1. Probiotic Spores of S. clausii SF174 Efficiently Adsorb Bromelain 

**Figure 1:**
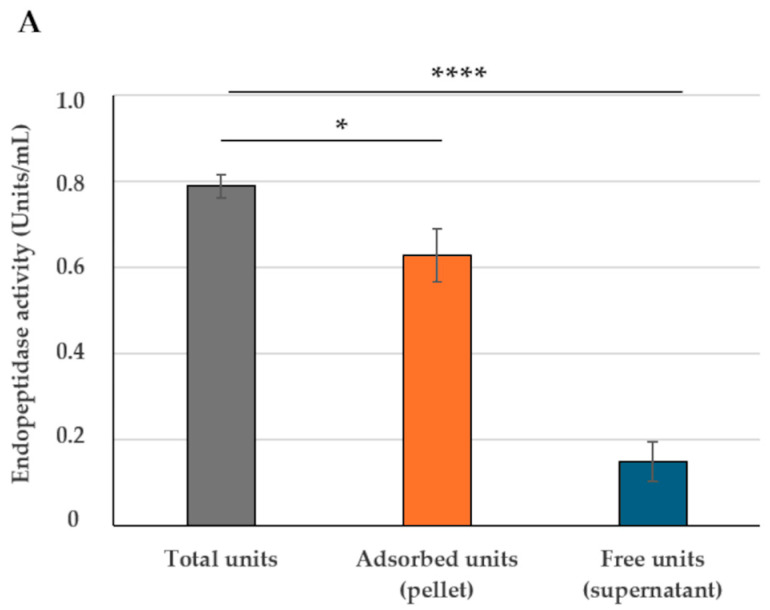

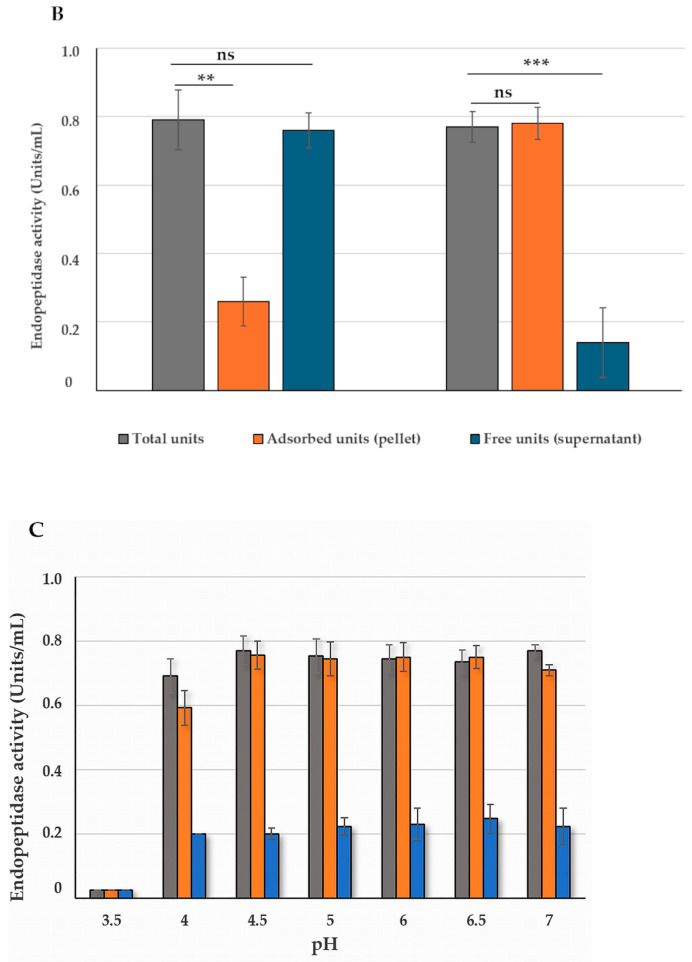
Bromelain endopeptidase activity before fractionation (total units, grey bars) and after fractionation by centrifugation, with pellet (adsorbed units, orange bars) and supernatant (Free units, blue bars). (**A**) Adsorption reaction performed in citrate buffer at pH 4.5. (**B**) Adsorption reaction performed in phosphate or acetate buffer at pH 4.5. (**C**) Adsorption reaction performed in acetate buffer at various pH values. The data represent the mean of three independent experiments, and the error bars are the standard error of the mean. *p*-values were calculated by using two-tailed *T*-test with ns indicating *p* > 0.05, * *p* < 0.05, ** *p* < 0.01, *** *p* < 0.001, and **** *p* < 0.0001, respectively.

In order to evaluate the adsorption of bromelain on SF174 spores, the endopeptidase activity of bromelain was followed. In the used experimental conditions (Materials and Methods), commercial pure bromelain had a linear kinetic of proteolytic activity between 10 and 100 μg/mL (Supplementary Material Figure S1). Up to 2.0 × 10^9^ free spores (without bromelain) of the SF174 strain showed only a minimal background of proteolytic activity (Supplementary Material Figure S2). Based on these observations, all further adsorption experiments were performed by using 1.0 × 10^9^ purified spores at conditions of enzyme linearity (50 μg/mL). As previously described for *B. subtilis* spores [20], bromelain was reacted with spores in citrate buffer at pH 4.5 for 1 h at 37 °C. An aliquot of the reaction mixture was assayed for protease activity to determine the total activity, while the remaining part was fractionated by centrifugation, and each fraction (pellet containing spore-adsorbed bromelain and supernatant containing free, not-adsorbed bromelain) was assayed independently. The adsorption rate (adsorbed protease units/total protease units) was 0.63, and over 75% of the proteolytic activity was found in the pellet fraction (Figure 1A), thus suggesting that most of the bromelain was adsorbed to the spore.

In order to improve the adsorption reaction, various buffers and pH conditions were tested. With respect to what was observed in the citrate buffer at pH 4.5 (Figure 1A), the proteolytic activity of the spore-adsorbed bromelain was lower in phosphate and higher in acetate buffer at pH 4.5 (Figure 1B). Indeed, the adsorption rates were 0.32 and 1.00 in phosphate or acetate buffer, respectively, indicating the latter as the best of the tested conditions for the interaction between SF174 spores and bromelain. 

Then, the acetate buffer was selected to test the adsorption rate at various pH conditions. As reported in Figure 1C, at pH values between 4.5 and 6.5, the activity of the spore-adsorbed bromelain resulted in similar adsorption rates in the range of 0.98 and 1.00 and slightly higher than pH 4.0 or 7.0, even if no statistical differences were observed (*p* > 0.05). In the experiments of Figure 1B,C, the activity of adsorbed plus not adsorbed bromelain (orange and blue bars, respectively) was higher than the total activity measured before fractionation (grey bars in the figures). This discrepancy is not surprising since an increase of activity of the spore-adsorbed enzyme with respect to the free form has been reported previously [20].

In acetate buffer at pH 4.5, over 95% of the endopeptidase activity was associated with the spore fraction, and all further adsorption experiments, except when stated differently, were conducted in those conditions.

### 2.2. Spore-Adsorbed Bromelain Is Stable at Simulated Gastric Conditions and Released at Simulated Intestinal Conditions

**Figure 2:**
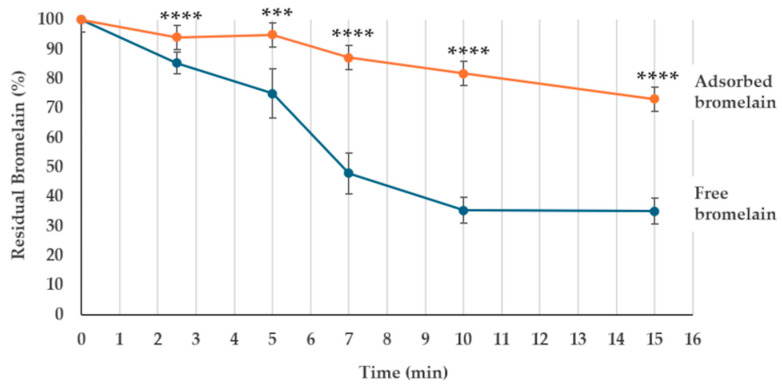
Endopeptidase activity of free (blue line) and spore-adsorbed (orange line) bromelain after various times of exposure at SGF at pH 2.5. The data are reported as a percentage of the bromelain activity, considering as 100% the activity before incubation at SGF conditions. The data represent the mean of three independent experiments, and the error bars are the standard error of the mean. *p*-values were calculated by using a two-tailed *T*-test with *** and **** indicating *p* < 0.001 and *p* < 0.0001, respectively.

Bromelain is unstable when exposed to gastric conditions, characterized by high acidity and the presence of bile salts [26,34]. To evaluate the stability of spore-adsorbed bromelain, the same amounts (50 μg) of free and spore-adsorbed bromelain were exposed to simulated gastric fluid (SGF) at pH 2.5 [29] for various times. As shown in Figure 2, while the free enzyme was rapidly degraded and less than 40% of the original proteolytic activity was detected after 15 min of SGF treatment, the spore-adsorbed enzyme was slowly degraded and over 70% of the original activity was detected after 15 min of treatment.

**Figure 3:**
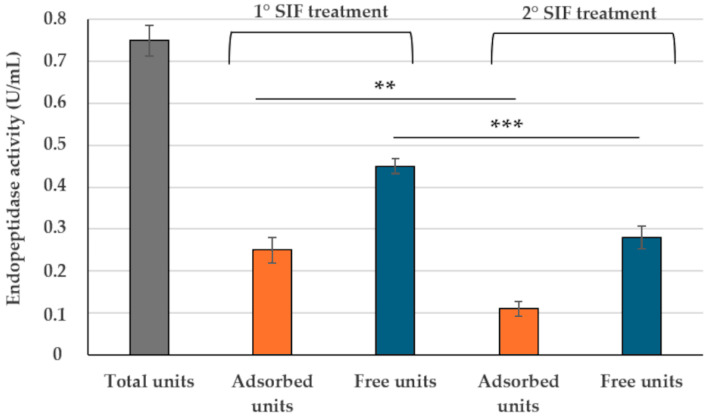
Endopeptidase activity of bromelain before fractionation (total units, grey bar) and after SIF treatments and fractionation leading to the pellet (adsorbed enzyme, orange bars) and the supernatant (free enzyme, blue bars). The data represent the mean of three independent experiments, and the error bars are the standard error of the mean. *p*-values were calculated by using a two-tailed *T*-test with ** and *** indicating *p* < 0.01 and *p* < 0.001, respectively.

When 0.75 Units/mL of spore-adsorbed bromelain was suspended in a simulated intestinal fluid (SIF) [29] and then fractionated by centrifugation as described above, over 65% (0.45 Units/mL of the initial 0.75 Units/mL) of the bromelain endopeptidase activity was released from the spore surface and found in the supernatant fraction (Figure 3). When a second SIF treatment was performed on the pellet fraction obtained from the first SIF treatment, all endopeptidase units still adsorbed to spores (0.25 Units/mL) were totally released (Figure 3). Indeed, the spore-bound endopeptidase activity observed after the second SIF treatment corresponded to that observed with the spore alone (Supplementary Material Figure S3). 

Altogether, results so far reported suggest that spore-adsorbed bromelain can safely cross the gastric barrier (Figure 2) and that in the intestine the adsorbed bromelain is released from the spore surface in an enzymatically active form (Figure 3).

### 2.3. Spore-Adsorbed Bromelain Is Released in an Active Form upon Spore Germination

In the intestine, spores germinate, allowing the temporary colonization of that habitat by germination-derived cells [10,15]. Spore germination involves the hydration of the spore cytoplasm, and the consequent increase of the spore volume is made possible by the physical disruption of the spore surface layers [14]. This raises the question of the fate of heterologous molecules adsorbed to spores upon the induction of germination.

**Figure 4:**
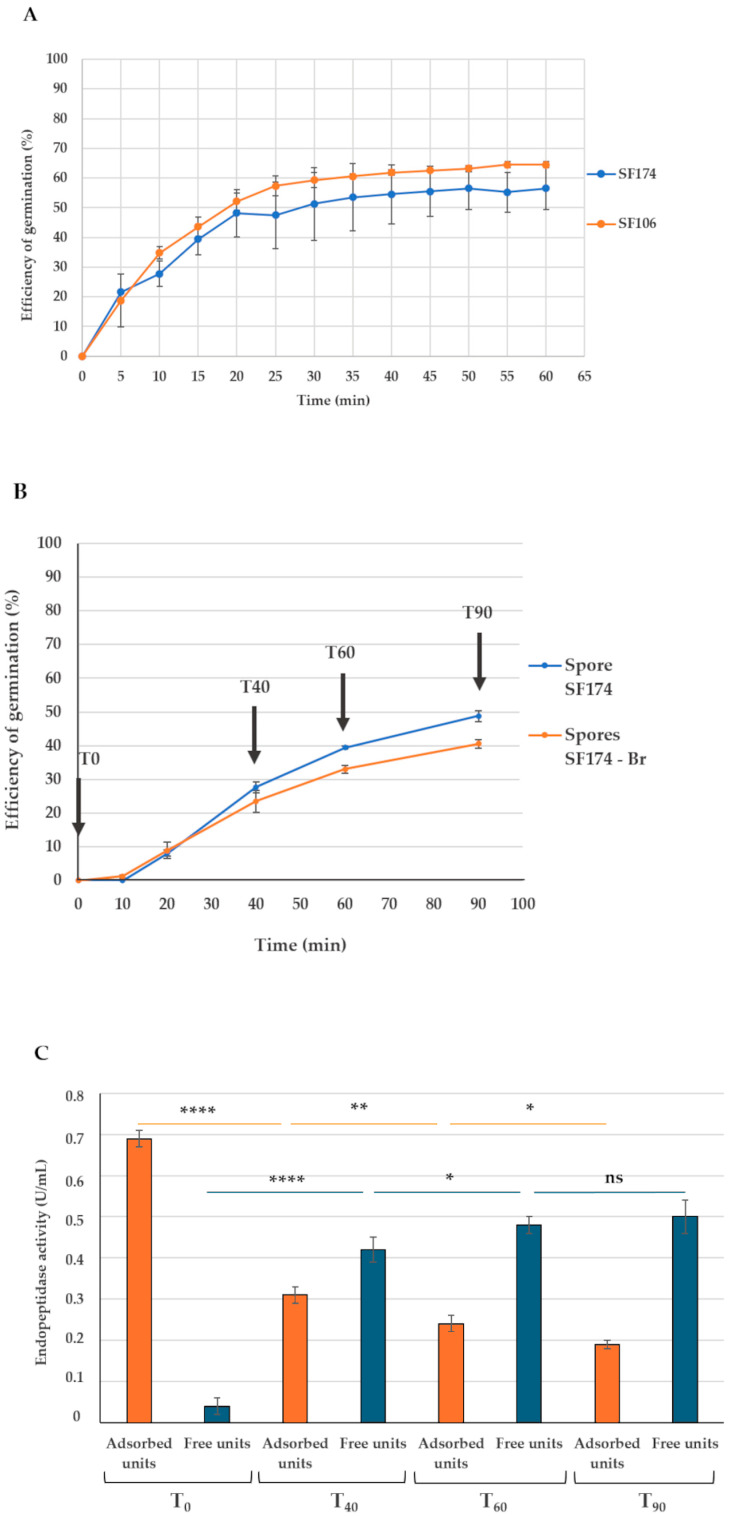
(**A**) Efficiency of germination monitored by OD loss of *B. subtilis* SF106 (orange line) and *S. clausii* (blue line) spores (1 × 10^7^). (**B**) Efficiency of germination monitored by OD loss of *S. clausii* spores (1 × 10^9^ spores) adsorbed (orange line) or not (blue line) with bromelain. The arrows indicate the time of collection of samples for panel C. (**C**) Endopeptidase activity of bromelain at the induction of germination (T0) and at the indicated times after the induction of germination. Samples of each time point were fractionated, and fractions were independently assayed: pellet (adsorbed enzyme, orange bars) and supernatant (free enzyme, blue bars). The data represent the mean of three independent experiments, and the error bars are the standard error of the mean. *p*-values were calculated by using a two-tailed *T*-test with ns indicating *p* > 0.05, * *p* < 0.05, ** *p* < 0.01, and **** *p* < 0.0001, respectively.

To answer this point, the efficiency of germination of SF174 spores was evaluated by following the OD_580nm_ decrease of a spore suspension, as previously developed for *Bacillus subtilis* spores [35]. As described in Section 4, purified SF174 spores (1 × 10^7^) were induced to germinate by the presence of L-asparagine, and the decrease of OD_580nm_ was followed over time. Since this protocol was originally established for *B. subtilis* and never tested for *S. clausii*, preliminary experiments were performed to compare the germination of SF174 spores with those of a *B. subtilis* strain (SF106) [31]. As shown in Figure 4A, using the tested protocol, SF174 (blue line) and SF106 (orange line) showed a similar response to the germinant L-asparagine, indicating that the procedure developed for *B. subtilis* can be reliably used also for SF174 spores. 

Then, the same germination procedure was scaled up to use the same amount of spores used for the adsorption reaction, and the efficiency of germination of spores alone and of bromelain-adsorbed spores was compared. As reported in Figure 4B, the germination of a large amount of spores (1 × 10^9^ in panel B of Figure 4) was slower and slightly less efficient than that observed with fewer spores (1 × 10^7^, panel A of Figure 4). The presence of bromelain had only a minor effect on the efficiency of spore germination (Figure 4B).

To evaluate whether upon germination the adsorbed bromelain was released in an active form, germinating spores were collected before the induction of the germination and 40, 60, and 90 min after (arrows in Figure 4B); the fractions were then separated by centrifugation, and the two fractions were tested for the endopeptidase activity, as described above. As shown in Figure 4C, at subsequent times from the induction of germination, the enzymatic units associated with the spore fraction (orange bars) gradually decreased while those found in the supernatant fraction (blue bars) gradually increased, suggesting that during germination the spore-adsorbed bromelain is released from the spores in an active form.

### 2.4. The Antioxidant Activity of SF174 Spores Is Maintained upon Bromelain Adsorption

**Figure 5:**
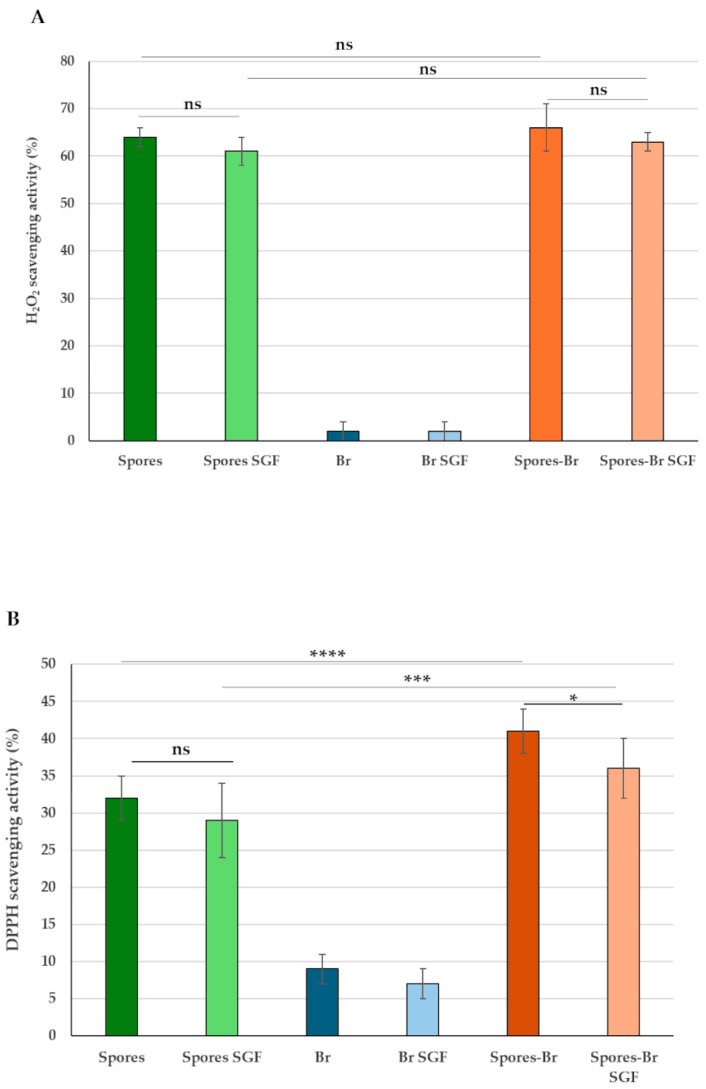
Antioxidant activity of spores (dark green bars), bromelain (dark blue bars) and spore adsorbed bromelain (dark orange bars). Light bars report samples analyzed after SGF treatment. (**A**) Hydrogen peroxide and (**B**) free radicals scavenging activities. The data represent the mean of three independent experiments, and the error bars are the standard error of the mean. *p*-values were calculated by using a two-tailed *T*-test with ns indicating *p* > 0.05, * *p* < 0.05, *** *p* < 0.001, and **** *p* < 0.0001, respectively.

SF174 spores are characterized by a strong antioxidant activity [30], while bromelain has only modest *in vitro* activity when compared to vitamin C [36]. Spores, free bromelain, and spore-adsorbed bromelain were analyzed for their antioxidant activity measured as the ability to scavenge H_2_O_2_ or free radicals (DPPH), as previously reported [30]. In our experimental conditions, 1.0 × 10^9^ spores of SF174 exhibited antioxidant activity and were able to efficiently scavenge H_2_O_2_ or free radicals (DPPH assay) (dark green bars in Figure 5), while the activity of 50 μg of pure bromelain was either not detectable (H_2_O_2_ scavenging) (Figure 5A) or very low (DPPH scavenging) (dark blue bar in Figure 5B). The antioxidant activity of 1.0 × 10^9^ spores was maintained (H_2_O_2_ scavenging) or increased (DPPH scavenging) when 50 μg of pure bromelain was adsorbed (dark orange bars in Figure 5), suggesting that spores and bromelain were synergically acting in scavenging free radicals (Figure 5B).

The antioxidant activity was also tested in the presence of SGF. To this aim, spores, free bromelain, and spore-adsorbed bromelain were incubated for 15 min in SGF as described above, and the antioxidant activity was tested. As shown in Figure 5, both the H_2_O_2_ and DPPH scavenging activities were not significantly altered by the SGF treatment (light vs. dark bars). Spore-adsorbed bromelain showed a slightly higher DPPH scavenging activity than spores alone also after the SGF treatment (light green bar vs. light orange bar in Figure 5B). 

### 2.5. SF174 Spores Efficiently Adsorb Food-Grade Bromelain

**Figure 6:**
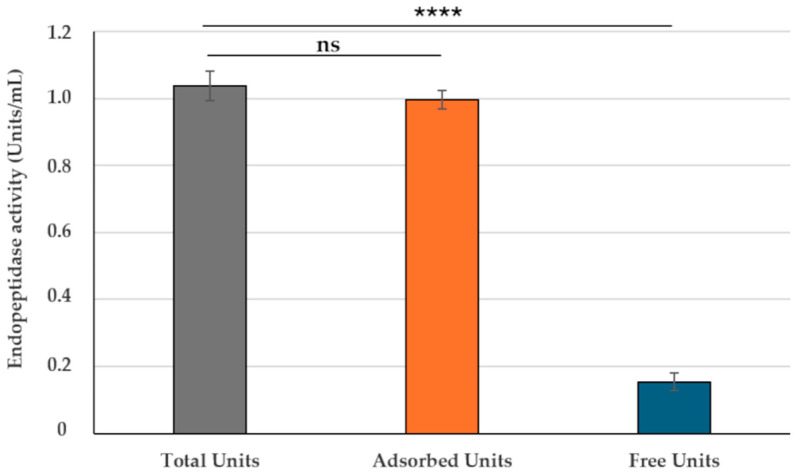
Endopeptidase activity of food-grade bromelain before fractionation (grey bars) or after fractionation by centrifugation, with pellet (adsorbed enzyme, orange bars) and supernatant (free enzyme, blue bars). *p*-values were calculated by using a two-tailed *T*-test with ns indicating *p* > 0.05 and **** *p* < 0.0001, respectively.

In order to evaluate the possibility of developing a prototype of spore-adsorbed bromelain for human use, commercial preparation of food-grade bromelain was tested. In the experimental conditions used (Section 4), food-grade bromelain had a linear kinetic of proteolytic activity between 0.5 and 2.5 mg/mL (Supplementary Material Figure S3). Based on this, 2 mg/mL of bromelain was reacted with 1.0 × 10^9^ SF174 spores in acetate buffer at pH 4.5 and assayed for protease activity, as described above. Upon fractionation by centrifugation, about 90% of the proteolytic activity was found in the pellet fraction, suggesting that most of the bromelain was adsorbed on the spore (Figure 6). 

Results of Figure 6 indicate that the food-grade preparation of bromelain behaves similarly to the research-only enzyme with respect to its interaction with SF174 spores.

## 3. Discussion 

The use of probiotics to support and improve human health has long been accepted, and a large variety of probiotic-based products are available on the market [1,2,3,4,5]. More recent is the idea of using probiotics for the oral delivery of healthy molecules, and only limited attention has been, so far, dedicated to such functional probiotics [16,19]. This study focuses on the development of a novel functional probiotic based on spores of the well-characterized probiotic strain SF174 adsorbed with the beneficial molecule bromelain.

The adsorption of heterologous molecules on bacterial spores has been characterized in detail and recently reviewed [19]. Heterologous molecules bind to spores through a combination of electrostatic and hydrophobic interactions without the formation of covalent bonds with specific spore surface components [37,38]. Also, based on their size, some heterologous molecules cross the outermost spore layers [39], infiltrating through pores present on the spore surface [19]. This is probably one of the reasons for the observed stabilization and protection of the adsorbed molecules [20]. Spore adsorption has been mainly used to display and deliver antigens [19] and enzymes [20] for applications as oral vaccines or recyclable biocatalysts, respectively.

A relevant result of this study is that bromelain is efficiently adsorbed on SF174 spores and that its endopeptidase activity is maintained and stabilized by the spore-adsorbed enzymes. Since the bromelain beneficial properties are based on the endopeptidase activity of the molecule [21], this result implies that SF174 spores, which do not have relevant endopeptidase activity, are functionalized by the interaction with bromelain, acquiring an additional beneficial property typical of the adsorbed molecule.

Ingested spores are known to germinate and perform some cycles of vegetative growth in the intestine, thus colonizing that habitat and exerting their probiotic roles [10,15]. Results reported here indicate that upon bromelain adsorption, SF174 spores are still able to germinate *in vitro*. This is an additional relevant result suggesting that SF174 spores, when adsorbed with bromelain, maintain their probiotic roles due to the proliferation of germination-derived cells in the intestinal district. 

Upon germination, the spore-displayed molecules are released in an enzymatically active form. This result, together with the observations that bromelain is protected against gastric conditions and released at intestinal conditions, proposes the spore of SF174 as a valuable carrier for the delivery of bromelain to the intestinal mucosal surfaces, where the beneficial action of bromelain will be reinforced by the well-characterized probiotic properties of SF174 spores [29,30,31,32,33]. 

When food-grade preparation was used, 2 mg of bromelain was adsorbed to 1.0 × 10^9^ spores. Although over 90% of the heterologous molecules were adsorbed to spores, the amount of bromelain delivered with a single dose of 1.0 × 10^9^ spores (approx. 1.8 mg) is far less than the amount contained in a commercially available capsule of free bromelain (45–90 mg) [40]. This is a clear limitation of the possible use of spores to deliver bromelain; however, a series of approaches could be attempted to improve the efficiency of the system. For example, the number of spores used for the adsorption reaction could be increased by 20-fold, since common spore-based probiotics for human use contain up to 2.0 × 10^10^ spores. In addition, the 2 mg used in this study for spore adsorption is not the maximal amount of bromelain potentially adsorbed and it is possible that spores of different probiotic strains may be more efficient in adsorbing heterologous molecules. All these possible approaches represent challenging new routes of future research plans aimed at translating the presented research results into a commercial product.

In spite of the limitations reported above, the observation that commercially available, food-grade bromelain is efficiently adsorbed on spores opens to the use of probiotic spores carrying bromelain as a novel functional probiotic. 

## 4. Materials and Methods

### 4.1. Induction of Sporulation and Spore Purification

*Sochuella clausii* SF174 was grown in Luria–Bertani medium (Difco Laboratories, New York, NY, US) at 37 °C in aerobic conditions. Sporulation was induced in Difco Sporulation (DS) medium (Difco, Detroit, MI, USA) by the exhaustion method [41]. After a 48 h incubation at 37 °C under vigorous shaking, spores were harvested by centrifugation (15 min; 9500 rpm) (SL8 Small Benchtop Centrifuge, Thermo Electron LED GmnH, Osterode am Harz, Germany), resuspended with 20 mL of 50 mM Tris-HCl (Tris base, Fisher, BioReagents^TM^, Waltham, MA, USA) pH 7.2 containing 50 µg/mL of lysozyme (L6876, Sigma Aldrich, San Louis, MO, USA), and incubated for 1 h at 37 °C under shaking. The spores were washed by adding a volume of sterile distilled water, and the suspension was centrifuged to obtain the pellet. It was resuspended with 20 mL of 0.05% sodium dodecyl sulfate (SDS, A1115636, Thermo Fisher Scientific Inc., Waltham, MA, USA) to degrade any proteins released from the vegetative cells and centrifuged. The spore pellet was washed three times with sterile distilled water, resuspended in a minimal volume of water, and stored at 4 °C, as previously reported [41]. Spore count and purity were determined by microscopy analysis. Spore counts by using a Petroff chamber (depth 0.02 mm) after dilution with pure distilled water. At least five large squares consisting of 16 small squares of four different chambers were counted. Spores were considered pure when less than 10% of vegetative cells were observed by microscopy analysis, as previously reported [41].

### 4.2. Bromelain Adsorption 

Commercial preparations of bromelain were used. Bromelain for research-use only (B4882, Sigma Aldrich, San Louis, MO, USA) or food-grade bromelain (020450, Farmalabor, Canosa di Puglia (BT), Italy). The adsorption of bromelain (B4882, Sigma-Aldrich^®^, San Louis, MO, USA) on SF174 spores was performed by incubating bromelain (typically 50 µg for the research-only product) with purified spores (typically 1 × 10^9^ CFU) in a final volume of 200 µL of 30 mM sodium acetate buffer pH 4.5 for 1 h at room temperature under shaking. To determine the total activity, the entire volume of the reaction mixture was assayed for protease activity (total units). The sample was fractionated by centrifugation (3 min at 9000 rpm) (MicroCL 21R Microcentrifuge, Thermo Electron LED GmnH, Osterode am Harz, Germany), and both the pellet containing spore-adsorbed bromelain (adsorbed units) and the supernatant containing the not-adsorbed bromelain (free units) were independently assayed.

### 4.3. Protease Activity of Bromelain

Bromelain was assayed as previously reported [42]. In brief, a reaction mixture containing 200 µL of 3% casein (C7078, Sigma Aldrich, San Louis, MO, USA), 0.2 M sodium phosphate buffer pH 7.0, and 200 µL of enzymatic solution was incubated at 37 °C for 10 min in a test tube. Exactly 1.2 mL of 2% trichloroacetic acid (A11156-36, Thermo Fisher Scientific Inc., Waltham, MA, USA) was added to stop the reaction, and the tubes were allowed to stand for 10 min. The mixture was centrifuged, and 200 µL of the supernatant fractions was transferred to a new tube. Then, 600 µL of 7.5% sodium carbonate (L13098-36, Thermo Fisher Scientific Inc., Waltham, MA, USA) solution was added, followed by 200 µL of Folin–Ciocalteau reagent (F9252, Sigma-Aldrich^®^, San Louis, MO, USA). Absorbance was measured at 660 nm after color development for 30 min. A control sample was prepared by adding the same quantity of the enzyme to the substrate after the reaction had been stopped. One unit of bromelain activity was defined as the amount of enzyme that liberated 1 μmol of tyrosine per minute under the assay conditions, and the protease activity was calculated by applying the following equation:

$$\mathrm{Protease}\;\mathrm{activity}\;\left(\frac{U}{\mathrm{mL}}\right)=\frac{\mathrm{Tyrosine}\;\mathrm{concentration}\left(µ\mathrm{mol}\right)\times \mathrm{volume}\;\mathrm{of}\;\mathrm{reaction}\;\mathrm{solution}\;\left(\mathrm{mL}\right)}{\mathrm{volume}\;\mathrm{of}\;\mathrm{crude}\;\mathrm{enzyme}\;\left(\mathrm{mL}\right)\times \mathrm{volume}\;\mathrm{of}\;\mathrm{sample}\;\mathrm{incuvette}\;\left(\mathrm{mL}\right)\times \mathrm{reaction}\;\mathrm{time}\;\left(\min\right)}$$

### 4.4. Hydrogen Peroxide Scavenging Assay

The hydrogen peroxide stability was measured by following absorbance at 240 nm of 0.036% (*w*/*w*) fresh hydrogen peroxide (H1009, Sigma-Aldrich^®^, San Louis, MO, USA) solution [50 mM of potassium phosphate buffer, pH 7.0; 0.036% (*w*/*w*) H_2_O_2_] at room temperature for 30 min.

Quantitative determination of H_2_O_2_ scavenging activity of spores, free bromelain, and spore-absorbed bromelain was measured by the loss of absorbance at 240 nm, as previously described by Beers and Sizer [43].

Briefly, spores, free bromelain, and absorbed bromelain samples were prepared at different concentrations by solubilizing them in fresh hydrogen peroxide solution. After 30 min of reaction, the samples containing spores only (1 × 10^9^) and spore-absorbed bromelain were centrifuged for 3 min at 9000 rpm to remove the spores, and the obtained supernatant was tested for H_2_O_2_ concentration measurements. The percentage of peroxide removed was calculated as follows:

$$\%\;H_{2}O_{2}\;\mathrm{scavenging}=1-\frac{{\mathrm{Abs}}_{240}\;\mathrm{sample}}{{\mathrm{Abs}}_{240}\;\mathrm{standard}}$$

where “Abs_240_ sample” is the absorbance of the reacted mixture of hydrogen peroxide solution with the extract sample and “Abs_240_ standard” is the adsorbance of the same mixture without the extract sample.

### 4.5. DPPH Assay

The α,α-diphenyl-β-picrylhydrazyl (DPPH, Thermo Fisher Scientific Inc., 044150.03) free radical scavenging method was used to evaluate the potential antioxidant activity of 1 × 10^9^ spores, free bromelain, and absorbed bromelain [44]. The samples were dissolved in sodium acetate buffer pH 4.5, and then 0.1 mM of DPPH dissolved in 100% methanol, at a ratio of 1:1, was added. As an internal standard and for comparison to published methods, was tested the antioxidant activity compounds ascorbic acid (vitamin C). The reaction was allowed to proceed for a maximum time of 30 min at room temperature in the dark and followed at 517 nm. The DPPH free radical scavenging activity was calculated according to the following equation:

$$\%\;\mathrm{DPPH}\;\mathrm{scavenging}=1-\frac{{\mathrm{Abs}}_{517}\;\mathrm{sample}}{{\mathrm{Abs}}_{517}\;\mathrm{standard}}$$

where “Abs_517_ sample” is the absorbance of the reacted mixture of DPPH solution with the extract sample and “Abs_517_ standard” is the adsorbance of the same mixture without the extract sample.

### 4.6. Gastric Condition

The samples containing either the adsorbed-bromelain or free bromelain were treated in an SGF solution containing 10 mM of HCl and 1 mg/mL of pepsin (P7000, Sigma Aldrich, San Louis, MO, USA) at pH 2.5 [29]. Specifically, the adsorbed bromelain (pellet fraction) was resuspended in 200 µL of the SGF solution while the free enzyme was directly solubilized in the SGF solution. The enzymatic proteolytic activity was followed at different time points up to fifteen minutes.

### 4.7. Intestinal Conditions 

The reaction mixture, constituted by spores and the attached bromelain, was rinsed for 10 min in simulated intestinal fluids (SIFs) containing 0.2% bile salts (LP0056, Thermo Fisher Scientific Inc., Waltham, MA, USA) and 1 mg/mL of pancreatin (P7545, Sigma Aldrich, San Louis, MO, USA) at pH 6.8 for three times at 37 °C [29]. The proteolytic activity of both pellet and supernatant was assayed after every wash.

### 4.8. Induction of Germination

Purified SF174 and SF106 spores (1 × 10^7^) were diluted in a 10 mM Tris-HCl (pH 8.0) buffer containing 1 mM D-(+)-Glucose (Thermo Fisher Scientific Inc., A16828-36), 1 mM of fructose (F-1950-50, Thermo Fisher Scientific Inc., Waltham, MA, USA), and 10 mM of KCl (BP366, Thermo Fisher Scientific Inc., Waltham, MA, USA). After 15 min at 37 °C, germination was induced by adding 10 mM of L-asparagine (Thermo Fisher Scientific Inc., B21473), and the optical density at 580 nm (OD_580_) was measured at 5 min intervals up to 1 h [35]. To test 1 × 10^9^ spores, the protocol was modified by increasing ten-fold the concentrations of KCl, glucose, and fructose and five-fold the concentration of L-asparagine. The decrement (%) was obtained by following the reduction of Abs_580_ at different times from the induction of germination (T0). 

## Data Availability

Dataset available on request from the authors.

## Supplementary Materials

The supporting information can be downloaded at https://www.mdpi.com/article/10.3390/ijms26030942/s1.
