# Supplementary material for: Probiotic Spores of Shouchella clausii SF174 and Displayed Bromelain Show Beneficial Additive Potential
Source: Int J Mol Sci. 2025 Jan 23;26(3):942. doi: 10.3390/ijms26030942 (PMC11817855; doi:10.3390/ijms26030942)
Supplement: Supplementary file 1 [file ijms-26-00942-s001.zip › ijms-3394457-supplementary.pptx]

## Slide 1
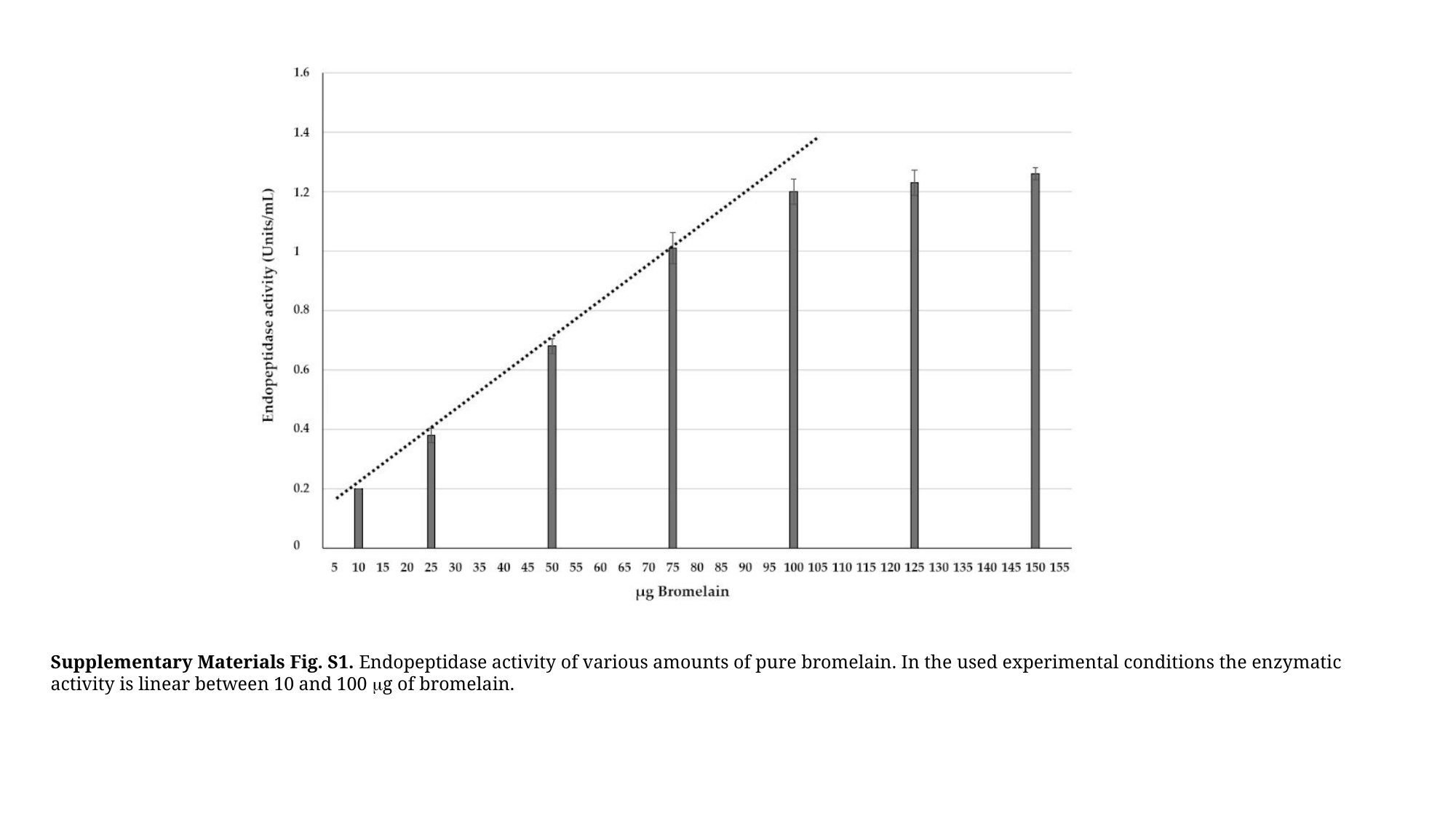

Supplementary Materials Fig. S1. Endopeptidase activity of various amounts of pure bromelain. In the used experimental conditions the enzymatic activity is linear between 10 and 100 mg of bromelain.

## Slide 2
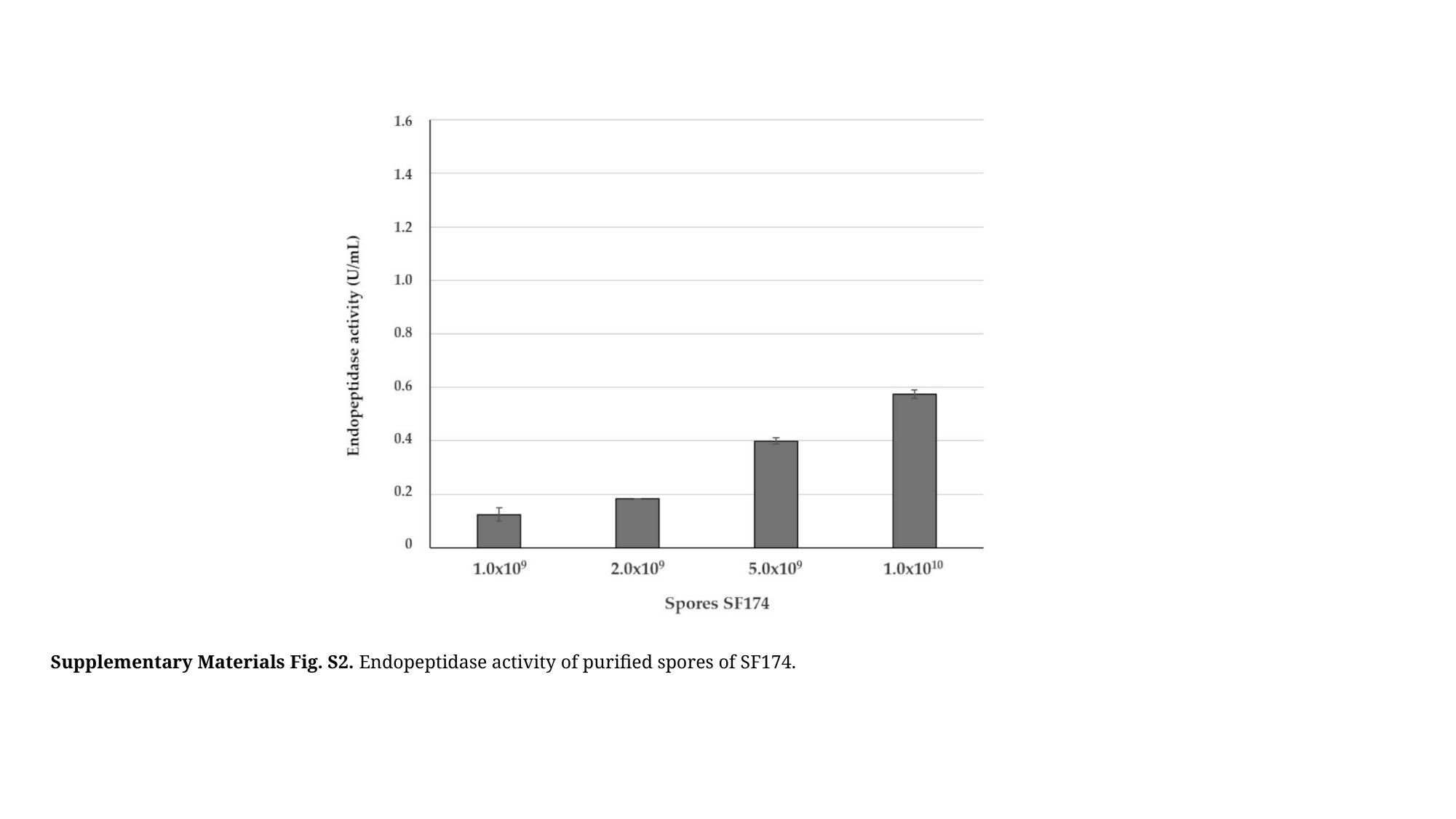

Supplementary Materials Fig. S2. Endopeptidase activity of purified spores of SF174.

## Slide 3
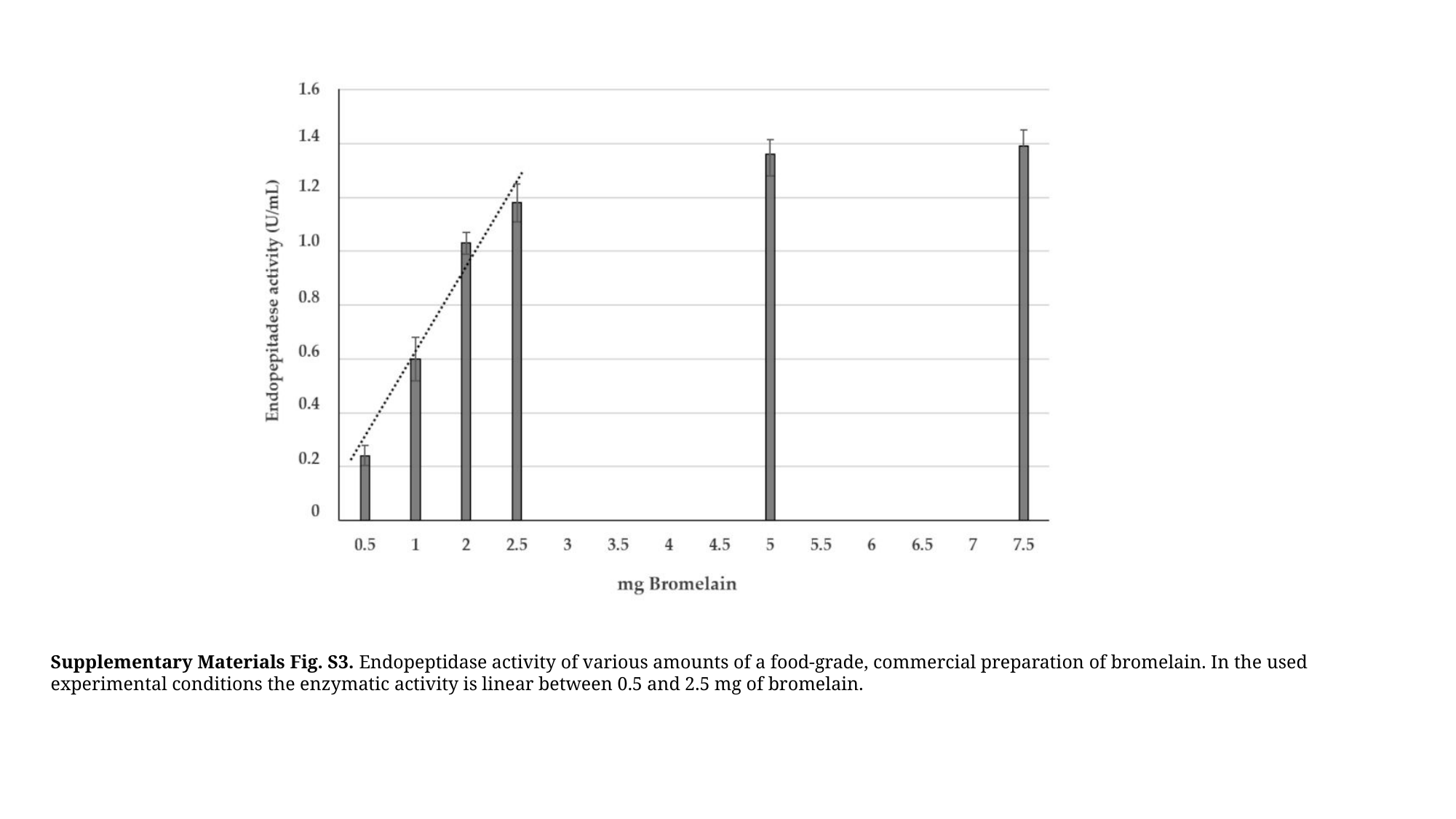

Supplementary Materials Fig. S3. Endopeptidase activity of various amounts of a food-grade, commercial preparation of bromelain. In the used experimental conditions the enzymatic activity is linear between 0.5 and 2.5 mg of bromelain.
